# Genetic Diversity Analysis of Brassica Yellows Virus Causing Aberrant Color Symptoms in Oilseed Rape

**DOI:** 10.3390/plants12051008

**Published:** 2023-02-23

**Authors:** Qi Peng, Wei Li, Xiaoying Zhou, Chengming Sun, Yan Hou, Maolong Hu, Sanxiong Fu, Jiefu Zhang, Jiban Kumar Kundu, Lei Lei

**Affiliations:** 1 Key Laboratory of Cotton and Rapeseed, Ministry of Agriculture, Institute of Industrial Crops, Jiangsu Academy of Agricultural Sciences, Nanjing 210014, China; 2 Institute of Life Sciences, Jiangsu University, Zhenjiang 212013, China; 3 College of Agronomy and Biotechnology, China Agricultural University, Beijing 100193, China; 4 Guizhou Rapeseed Institute, Guizhou Academy of Agricultural Sciences, Guiyang 550008, China; 5 Plant Virus and Vector Interactions-Centre for Plant Virus Research, Crop Research Institute, Drnovska 507/73, 161 06 Praha, Czech Republic; 6 Laboratory of Virology-Centre for Plant Virus Research, Institute of Experimental Botany of the Czech Academy of Sciences, Rozvojová 263, 165 02 Prague, Czech Republic

**Keywords:** BrYV, TuYV, oilseed rape, phylogenetic tree, incidence, leaf color

## Abstract

The emergence of brassica yellow virus (BrYV) has increasingly damaged crucifer crops in China in recent years. In 2020, a large number of oilseed rape in Jiangsu showed aberrant leaf color. A combined RNA-seq and RT-PCR analysis identified BrYV as the major viral pathogen. A subsequent field survey showed that the average incidence of BrYV was 32.04%. In addition to BrYV, turnip mosaic virus (TuMV) was also frequently detected. As a result, two near full-length BrYV isolates, BrYV-814NJLH and BrYV-NJ13, were cloned. Based on the newly obtained sequences and the reported BrYV and turnip yellow virus (TuYV) isolates, a phylogenetic analysis was performed, and it was found that all BrYV isolates share a common root with TuYV. Pairwise amino acid identity analysis revealed that both P2 and P3 were conserved in BrYV. Additionally, recombination analysis revealed seven recombinant events in BrYV as TuYV. We also attempted to determine BrYV infection by quantitative leaf color index, but no significant correlation was found between the two. Systemic observations indicated that BrYV-infected plants had different symptoms, such as no symptom, purple stem base and red old leaves. Overall, our work proves that BrYV is closely related to TuYV and could be considered as an epidemic strain for oilseed rape in Jiangsu.

## 1. Introduction

Oilseed rape (*Brassica napus* L.) is the second most important oil crop in the world. The occurrence of a plant virus disease would lead to both a decline in oilseed rape production and a deterioration in the oil quality of oilseed rape. Several viruses have been identified that have different symptoms and effects on oilseed rape. Among them, turnip yellows virus (TuYV) has caused huge yield losses in many countries, but it has long been overlooked because infected plants are asymptomatic or show nutrient deficiency symptoms [1,2]. TuYV, formerly known as beet western yellows virus (BWYV), belongs to the genus *Polerovirus*, family *Solemoviridae* [3]. Although genetic improvement to enhance resistance to TuYV has been pursued in Europe, this has not been widely implemented in Asia [4]. In contrast, brassica yellow virus (BrYV), which has about 80% nucleotide sequence identity with TuYV, has been discovered in Japan and South Korea and is increasingly causing damage to crucifer crops in China [5,6,7,8].

All members of the polerovirus are transmitted persistently, circulatively and non-propagatively by aphids and are characterized by phloem limitation [9]. The genome of BrYV contains a single positive RNA with a 5′-terminal modification of VPg. Based on host species and genome sequence information, BrYV has been divided into strains A, B and C [10]. Both types A and B contain a genome of 5666 nt in length and have nucleotide identity of 94.2–94.7% to each other. The genome of strain C has a length of 5678 nt and has a nucleotide identity of 93.4–94.8% compared to A and B [10]. Moreover, strains A and B mainly infected turnips and oilseed rape, while type C was mainly observed on radish and cabbage, respectively [10]. Recently, two BrYV isolates from radish were identified as a new strain, BrYV-R [11]. Poleroviruses use several strategies, including subgenomic RNA, shift frame, leaky scanning, read-through and non-ATG initiation [12]. As a result, ORF0 encodes RNA silencing suppressor (RSS), ORF1 encodes Pro-VPg by leaky scanning and P1 generates P1-P2 by frameshifting, which encodes RNA-dependent RNA polymerase (RdRp) [13,14,15,16]. On the subgenomic RNA, 3a and the movement protein (MP) are encoded by ORF3a and ORF4, which are involved in systemic and cell-to-cell movement, respectively [17,18,19]. The P3 and P3-P5 read-through proteins are the major and minor components of the viral capsid, respectively [20,21]. To date, BrYV research has focused on developing detection methods and identifying the functions of the viral proteins. However, the relationship between BrYV, which is common in Asia, and TuYV, which is common worldwide, remains controversial. In addition, the occurrence of BrYV on oilseed rape and the symptoms caused by the virus in the field, which are crucial for disease diagnosis, have not yet been sufficiently investigated.

Jiangsu Province (located on the lower reaches of the Yangzi River), is one of the most important oilseed rape growers in China. In the winter of 2020, a scale of oilseed rape in Yangzhong, Jiangsu Province, showed symptoms red discoloration. In this work, we combined RNA-seq with RT-PCR technologies and identified BrYV as the major viral pathogen responsible for the disease symptoms. We then investigated the occurrence of BrYV in the field and first systematically mapped the symptoms caused by BrYV in *B. napus*. We also investigated the correlation between leaf color and virus infection status. Furthermore, we cloned two nearly complete BrYV sequences and analyzed the phylogenetic relationships within the BrYV groups and between BrYV and TuYV based on nucleotide and amino acid sequences. The results show that there is a close relationship between BrYV and TuYV. In addition, BrYV is the predominant virus in Jiangsu, the infection of which can lead to the occurrence of disease symptoms in oilseed rape.

## 2. Results

### 2.1. BrYV Was the Most Important Viral Pathogen in Oilseed Rape in Yangzhong, Jiangsu

**Figure 1:**
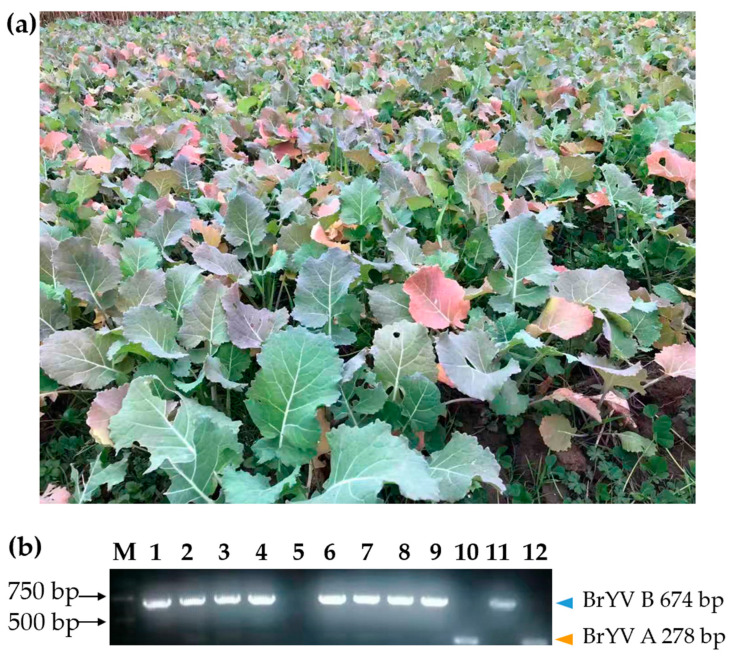
Symptoms of oilseed rape in the field and virus detection. (**a**) Oilseed rape showing the symptom of red leaves in Yangzhong in 2020. (**b**) RT-PCR based BrYV detection with primer pair BrYA484F/BrYB88F/BrYC257F/BrY761R, 278/674/505 bp. M stands for 100 bp DNA marker. Lanes 1 to 10 show 10 samples with red leaves collected in the field. The positive control of BrYV-B and BrYV-A plasmid in lane 11 and 12 was marked with an orange and blue triangle, respectively.

In the winter of 2020, red discoloration and nutrient deficiency-like symptoms were observed on a large number of oilseed rape plants, during a disease survey in Yangzhong, Jiangsu Province, China (Figure 1a). A preliminary estimate showed that 20–60% of the plants had significantly abnormal leaf color, irregularly distributed over 1500 square meters of oilseed rape field. Considering that oilseed rape had been grown on the plot for many years, but that the symptoms resembled a nutrient deficiency, the investigation into the causes of the symptoms was undermined. The assignment of the causal agents of these symptoms was investigated for the first time by RNA-seq analysis of the leaf samples. Sequencing results showed that several viral fragments, including BrYV (NC016038), TuYV (NC003743), BWYV (NC004756), cucurbit aphid-borne yellows virus (CABYV, NC003688), cucumber mosaic virus (CMV, NC002034), gay feather mild mottle virus (GMMV, NC012134), CABYV-associated RNA (CABYV-AR, NC026508) and BWYV-ST9 (NC004045), were present in the sample. Then, RT-PCR was performed to verify the results. However, only the fragments of BrYV, BWYV-ST9 and CABYV-AR were repeatedly detected and successfully identified by Sanger sequencing. In individual plants, nine out of ten symptomatic oilseed rape plants collected in Yangzhong were found to be infected with BrYV (Figure 1b). And 6 to 13 samples with similar symptoms from Nanjing in Jiangsu province were also identified as BrYV-positive.

To determine the incidence of viral diseases, field surveys were conducted in Yangzhong using this method. As shown in Table 1, the average BrYV infection rate was 32.04%, while the highest incidence was up to 52.94%. When counting the incidence by different BrYV strains, we found that both BrYV A and B could successfully infect oilseed rape independently or mixed (Table 1). Six plants in fields B and E were identified as TuYV-positive. However, the result of subsequent Sanger sequencing showed that these samples were still infected with BrYV and not TuYV. Although high-throughput sequencing found CMV and not turnip mosaic virus (TuMV), it could not be detected in the mixed pool or in individual samples. On the contrary, TuMV was found in the RT-PCR analysis during field diagnosis. In fact, TuMV was widespread in the field, with an average incidence of 24.27% (Table 1). In addition, three plants in fields B and E were positive for CABYV-AR and four plants in fields A and E were positive for BWYV-ST9. These results indicate that BrYV is the most important viral pathogen in Jiangsu oilseed rape, which can lead to different colored leaf symptoms.

### 2.2. BrYV Infection Causes Different Symptoms in the Field

**Figure 2:**
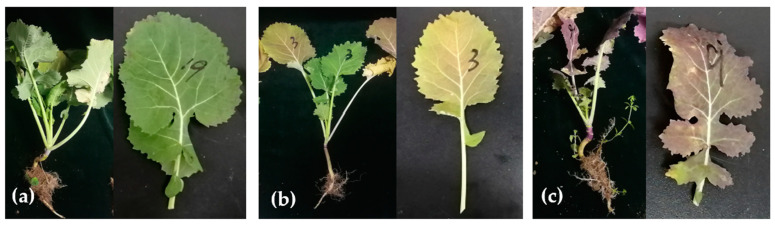
Typical BrYV symptoms occurring mainly in three types of plants in the field. (**a**) Type I, plant without significant symptoms but tested BrYV positive; (**b**) Type II, BrYV-infected plant with leaf yellowing; (**c**) Type III, BrYV-infected plant with conspicuous red or purple leaves.

We recorded the symptoms caused by BrYV based on the leaf surface. We divided all 33 BrYV-infected plants into three groups according to their leaf characteristics. Type I comprised nine plants that showed no visible, perceptible difference from healthy plants (Figure 2a and Supplementary Figure S1a). Five plants of type II showed yellowing symptoms on old leaves (Figure 2b, Supplementary Figure S1b). In addition, 19 plants with obviously red-colored old leaves were grouped together in III (Figure 2c, Supplementary Figure S1c). In this group, some plants had a whole reddish old leaf, such as A25. Some plants had uniform or local purple and red leaves, such as D12. Some had a reddish color at the leaf margin, such as C8. In addition, many plants had distinctly purple stem bases, such as C2 (Supplementary Figure S1c). BrYV-A, -B single infections and double infections showed no obvious differences in the oilseed rape. Based on these observations, we assumed that BrYV infection in oilseed rape could lead to complicated symptoms in the field.

### 2.3. The Relationship between Leaf Color and the Occurrence of the Virus

**Figure 3:**
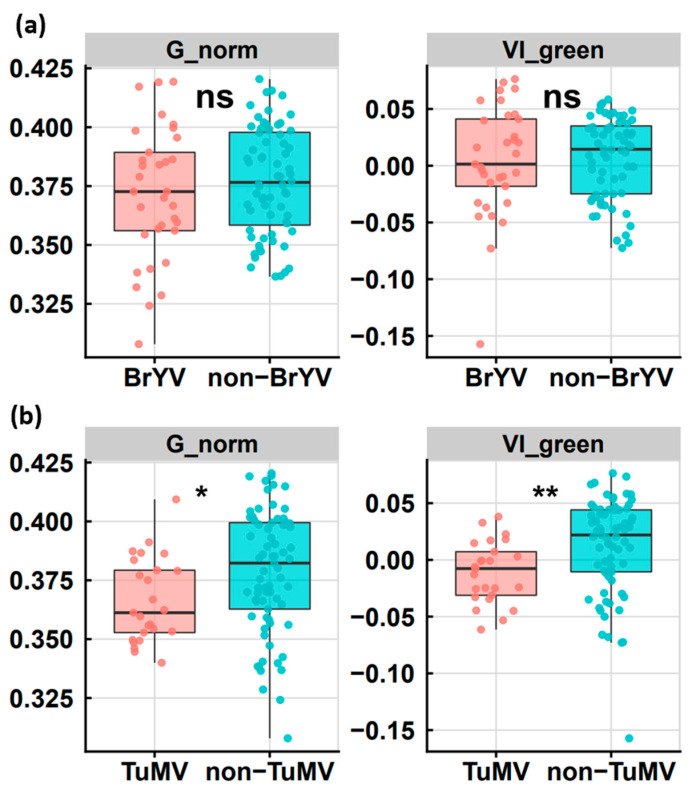
The comparison of leaf color values between virus-infected and non-infected plants. (**a**) The difference in leaf color values between BrYV-infected (pink) and non-infected (blue) plants. (**b**) The difference in leaf color values between TuMV-infected (pink) and non-infected (blue) plants. The leaf color values were estimated by the parameters G_norm (**left**) or VI_green (**right**). Student’s *t*-test was used for hypothesis testing. Single asterisk indicates a *p* value < 0.05 and double asterisks indicate a *p* value < 0.01.

TuYV infection could lead to nutrient deficiencies in oilseed rape [1]. Transgenic *Arabidopsis* with full-length BrYV-C also showed purple leaf symptoms [22]. To evaluate the relationship between leaf color and virus infection status, we first extracted the leaf appearance of samples from fields A to E and assessed the leaf color value according to the method. We then divided the samples into BrYV-infected and non-infected groups based on the results from RT-PCR and compared the leaf color values between the two groups. As can be seen in Figure 3a, although the average G_norm and VI_green of the BrYV-infected leaves were lower than those of the non-BrYV-infected leaves, no statistical significance was found in the color values between the two groups. In comparison, TuMV-infected leaves had a significantly lower color value than non-TuMV-infected plants for both the G_norm and VI_green parameters (Figure 3b). This suggests that TuMV may cause a more stable chlorosis symptom in oilseed rape. To exclude the influence of the field, we also repeated the analysis on five different fields. We found that field C had the highest values in both the G_norm and VI_green measurements, indicating a relatively low incidence of the virus infection (Supplementary Figure S2). In comparison, the results of the G_norm parameters did not agree well with the VI_green assessment in the other four fields.

A correlation analysis was then performed between the occurrence of the virus and the leaf color. The result shows that neither the occurrence of BrYV nor TuMV was relevant for the leaf color rating (Supplementary Table S1). However, the correlation between the occurrence of virus diseases and the G_norm or VI_green value was relatively high (Supplementary Table S1). Nevertheless, the correlation coefficients were not striking and were below 0.5, indicating that virus infection was only one of the causes of the chlorosis symptom in the field.

### 2.4. Phylogenetic Analysis of BrYV and TuYV Sequences 

**Figure 4:**
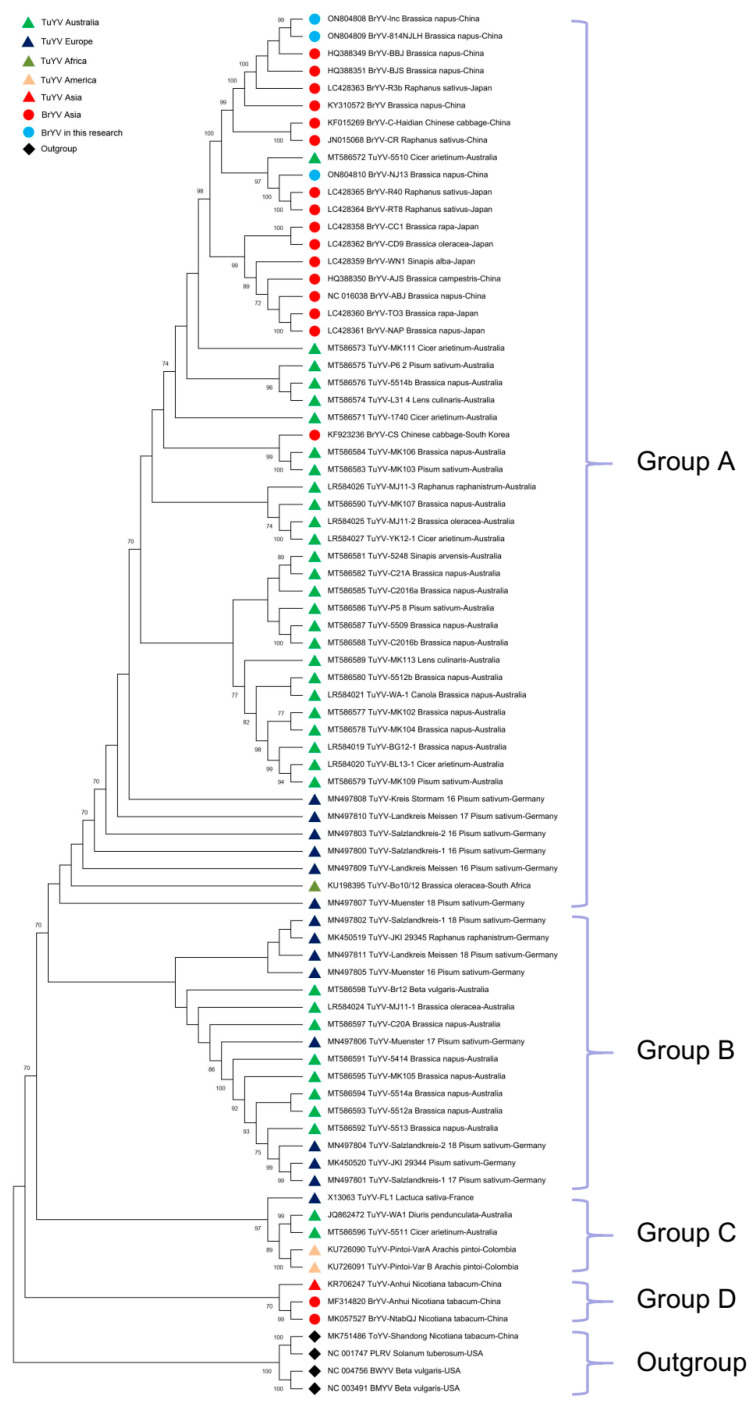
Phylogenetic tree constructed using the available nearly complete nucleotide genome sequences of BrYV isolates (*n* = 21) and TuYV isolates (*n* = 55). The phylogenetic analysis was derived with maximum likelihood (ML) based on the General Time Reversible (GTR) model, which was selected as the best model of nucleotide substitution based on the Bayesian Information Criterion (BIC) as implemented in MEGA X. Isolates are identified by their GenBank accession numbers, isolate name, host and geographical location (Supplementary Table S2). Bootstrap values of more than 70% (1000 bootstrap repeats) are indicated.

Based on contigs matching BrYV, a full-length sequence of BrYV Jiangsu was isolated and designated, BrYV-lnc (accession number ON804808). Specific primers corresponding to the BrYV-lnc sequence were then designed to amplify the full-length BrYV sequence (Methods). The result is an almost complete sequence clone, BrYV-814NJLH (accession number ON804809), obtained from the Yangzhong sample. Another nearly complete BrYV sequence, BrYV-NJ13 (accession number ON804810), was obtained from a sample from Nanjing. The BrYV sequences strongly resemble TuYV. To better understand the relationships within the BrYV isolates and the relationship between BrYV and TuYV, a phylogenetic analysis was performed. A phylogenetic tree was constructed based on the full-length nucleotide sequences of 21 BrYV isolates, 55 TuYV isolates and 4 outgroup species (BWYV, beet mild yellowing virus (BMYV), tobacco yellow virus (ToYV) and potato leafroll virus (PLRV)). The phylogenetic analysis tree divided all isolates into four main groups: A, B, C and D (excluding the outgroup, Figure 4). Group A included 33 TuYV isolates (26 from Australia, 6 from Germany and 1 from South Africa) as well as 3 Jiangsu BrYV isolates from this study and 16 previously reported BrYV isolates (7 from China, 8 from Japan and 1 from Korea). Among them are BrYV-lnc and BrYV-814NJLH, which belong to BrYV type B. Both have the highest similarity to the BrYV-BBJ isolation (Accession HQ388349.1) and have nucleotide sequence identity of 98.82% and 98.84%, respectively (Figure 4). BrYV-NJ13 was observed together with two isolates from radish and one TuYV isolate. The four sequences have formed an additional subgroup referred to as BrYV type R [11] (Figure 4). BrYV-NJ13 has the highest similarity to BrYV-R40 (accession number LC428365.1) and the nucleotide sequence identity between the two is 96.74%. Group B comprises 16 TuYV isolates (8 from Germany and 8 from Australia). Group C comprises five TuYV isolates (two from Colombia, two from Australia and one from France). Group D includes two BrYV and one TuYV isolate, but all originate from *Nicotiana tabacum* in China and differ from the hosts of the other isolates. For TuYV, the phylogenetic tree clearly shows high diversity (Figure 4). However, all BrYV isolates are included in the TuYV clade, although some TuYV sequences are distributed in the BrYV clades, such as TuYV-5510 (accession number MT586572) and TuYV-MK111 (accession number MT586573) (Figure 4).

### 2.5. Pairwise Analysis of the BrYV Proteins

To further investigate variation within BrYV groups, the seven BrYV-encoded proteins were analyzed separately. TuYV-FL1, which was far from BrYV due to nucleotide identity, served as a control (Figure 4). For the 5′ part of the genome, P0, P1 and P2 showed similar topology (Supplementary Figure S3a–c). BrYV-NAP, -TO3, -ABJ, -ABJ-NC, -CS, -WN1 and -AJS came together in all three trees. Meanwhile, BrYV-NJ13, -R40 and RT8 formed one group. BrYV-BBJ, -R3b, -China, -lnc, -814NJLH and -BJS formed a separate group in both P0 and P1, but were split into different groups in P2 (Supplementary Figure S3). P2 was the most conservative protein among BrYV isolates with amino acid (aa) identities between 97.3–100% (Table 2). Even compared to TuYV-FL1, the aa identities of BrYV P2 were 96.22–97.84%. BrYV P1 was more variable than P0 (91.97–100%), with an aa identity of 88.3–100%. However, the aa identity between BrYV P0 and TuYV-FL1 P0 was slightly lower than the ratio between BrYV P1 and TuYV-FL1 P1 (Table 2).

For the first protein encoded by the 3′ parts of the genome, P3 was the second conservative ORF in BrYV, whose aa identity was 94.06–100% (Table 2). The phylogenetic tree of P3a mainly contained three groups and the aa identity ranged from 82.22 to 100%. Among them, BrYV-TO3, -NAP and -Tas formed the first group. BrYV-RT8, -WN1, -CC1, -CD9, -R3b, -R40 and TuYV-FL1 formed the second group and the others formed the third group (Supplementary Figure S3e). The topology of phylogenetic tree P4 was similar to that of tree P3 (Supplementary Figure S3d,f). BrYV-Anhui, -NtabQJ, -R40, -RT8, -CS and TuYV-FL1 formed one group and the rest formed another group. Interestingly, the pairwise aa identity between BrYV and TuYV-FL1 was higher than that within BrYV in P1, P3, P3a and P4 (Supplementary Figure S3 and Table 2).

**Figure 5:**
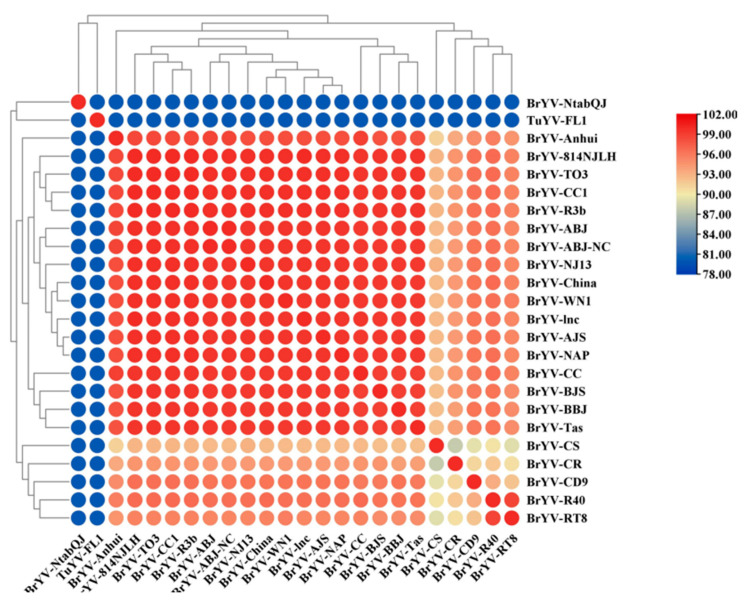
Pairwise amino acid analysis based on the phylogenetic tree of BrYV P5. The matrix shows the identity of amino acids (aa) between the different virus isolates. The order of the isolates was determined by the contralateral phylogenetic tree. The color of the dots indicates the pairwise amino acid identity.

The phylogenetic tree for P5 showed three main groups (Figure 5). BrYV-NtabQJ and TuYV-FL1 formed one group, BrYV-CS, -CR, -CD9, -R40 and -RT8 formed the second group. The rest of the sequences formed the third group. Previous studies have shown that P5 is the most variable protein in both BrYV and TuYV [8,23,24]. If BrYV-NtabQJ and TuYV-FL1 P5 were removed, the lowest aa identity of the remaining BrYV P5 could increase from 51.55 to 89.16, which would correspond to that of P0, P1 or P4 (Table 2).

The pairwise amino acid identities between BrYV-814NJLH or BrYV-lnc and BrYV, and between BrYV-NJ13 and BrYV were similar. In particular, P0, P2 and P3 were relatively well conserved with identity values above 93.17%. The pairwise identities in P1, P3a and P4 were intermediate, with values above 84.44%. In contrast, P5 had the highest deviation. The pairwise identity of BrYV-814NJLH with BrYV in P5 ranged from 57.11–100%, while the value between BrYV-NJ13 and BrYV ranged from 56.91–99.75% (Table 2).

### 2.6. Recombination Analysis

**Figure 6:**
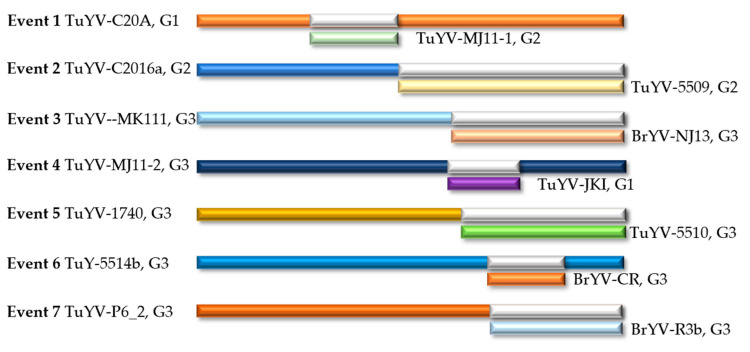
The schematic representation of the recombinants identified in this study. The long colored bars represent the genome of the recombinant. The shorter dark grey bars indicate the potential position of the recombinant, and the corresponding shorter colored bars indicate the donor fragment of the smaller parent. The abbreviation “G” after the train name stands for “group” according to the reference.

Recombination plays an important role in the evolution of viruses. BrYV is genetically closely related to TuYV, as shown by the phylogenetic tree. Next, we tried to detect recombinant events in TuYV and BrYV isolates to further investigate the relationship between the two. As a result, seven events had strong recombination signals that were repeatedly detected by RDP (R), GENECONV (G), BOOTSCAN (B), MAXCHI (M), CHIMAERA (C), SISCAN (S) and 3SEQ (T) methods with a *p*-value of less than 1 × 10^−13^ (Table 3) [25]. In addition, all events with unique breakpoint positions and with unique major and minor parents were determined (Figure 6, Supplementary Figure S4, Table 3).

In a previous report, TuYV sequences were classified into three groups based on the phylogenetic relationship of ORF5 [24]. Using the same criterion, we found that recombinant events 1 and 4 occurred in the intro groups and the other five events occurred in the inter groups (Figure 6). For event 1, according to the RDP, the start and end points of recombination were 1492 nt and 2663 nt of TuYV-C20A (accession number MT586597), respectively (Supplementary Figure S4 and Table 3). The recombinant TuYV-C20A belonged to group 1, while the smaller parent TuYV-MJ11-1 (accession number LR584024) belonged to group 2 (Figure 6). The predicted recombinant fragment of event 4 was located between 3316 nt and 4277 nt of TuYV-MJ11-2 (accession number LR584025, Supplementary Figure S4 and Table 3). The recombinant TuYV-MJ11-2 and the smaller parent TuYV-JKI (MK450520) belonged to group 3 and group 1, respectively (Figure 6). Event 2 was determined from 2668 nt downstream of TuYV-C2016a (accession number MT586585, Supplementary Figure S4 and Table 3). Both the recombinant TuYV-C2016a (accession number MT586585) and the smaller parent TuYV-5509 (accession number MT586587) belonged to group 2 (Figure 6). Events 3, 5, 6 and 7 all occurred in group 3 (Figure 6). Among them, the newly identified BrYV-NJ13 was determined to be the putative donor of event 3, which started from 3368 nt to the end of TuYV-MK111 (accession number MT586573, Table 3, Figure 6, Supplementary Figure S4).

## 3. Discussion

We found that the incidence of BrYV was the highest at 52.94%, and all the surveyed fields in Jiangsu were affected by the virus infection. Although TuMV was also frequently detected, its average incidence (24.27%) was lower than the average incidence of BrYV (32.04%). The high incidence of BrYV and the increasing area of occurrence indicate that BrYV is becoming an important pathogen of oilseed rape in Jiangsu. In addition to Jiangsu, BrYV has also been detected in several other oilseed rape growing regions, including Chuxiong in Yunnan and Chuangshun in Guizhou Province (Supplementary Figure S5). This indicates that BrYV is widespread in oilseed rape growing areas in both the upper and lower reaches of the Yangtze River. Occurrence of the virus was also recently reported in several Brassica species, including *Brassica napus*, in China in 2014–2018, with an average incidence of 17.0% and 11.6% in eight cruciferous species and oilseed rape, respectively [5]. Presumably, the incidence of BrYV is more common in *Brassica* crops in China, and further surveys may provide clear evidence of the spread of the virus and the resulting yield losses. Although the symptoms of BrYV, including yellowing, red and purple leaves and purple stem tips, are visible in the field, some plants may show latent symptoms or changes in leaf color resembling nutrient deficiency, leading to misinterpretation (Supplementary Figure S1a). This explains why the general leaf color analysis showed no significant difference between BrYV-infected and non-infected plants (Figure 3a) in the field. The use of image analysis is becoming increasingly important in the detection of diseases in crops. In this study, we attempted to qualify leaf color to determine the occurrence of viral diseases. Although the occurrence of BrYV or TuMV individual infections hardly correlates with leaf color, the total virus occurrence shows a relatively stronger correlation with the value of leaf color (Supplementary Table S1). This shows that virus infection does indeed have some influence on leaf color in the field. Overall, the implementation of image recognition for early and large-scale detection of virus diseases in oilseed rape or other field crops needs further improvement.

Compared to TuYV, BrYV has a relatively small host range, both in terms of geographical distribution and plant species. While TuYV has been found in Europe, Africa, Australia and Asia [1,24,26,27,28,29], BrYV has been found mainly in Asia, including China, Japan, South Korea and Australia [5,6,7,23]. TuYV has a stronger host adaptation and infects plant species from 24 plant families, including crops and weeds [30]. In comparison, BrYV has previously been detected mainly in crucifers or vegetables, and a recent study reported its occurrence in a Brassica weed [5,7,11,23]. In this study, BrYV infection was found to cause various symptoms related to leaf color, including yellowing, red or purple leaves and purple stem bases (Supplementary Figure S1). Overall, the symptoms caused by BrYV are very similar to those of TuYV, as described in the literature [1,31].

The sequence analysis in our results shows a close relationship between BrYV and TuYV. Based on amino acid identity, we found that TuYV-FL1, which was distant from BrYV, was grouped according to nucleotide identity (Figure 4). However, some proteins of TuYV-FL1 could not be distinguished from BrYV isolates by pairwise amino acid analysis, such as P1, P3, P3a and P4 (Table 2, Supplementary Figure S3). Amino acid sequence alignments showed that each protein of BrYV shared a common root with the corresponding TuYV protein, which was distinct from other poleroviruses, such as BWYV, BMYV or CABYV [11,24]. Furthermore, recombination analysis showed that there was genetic information exchange between TuYV and BrYV (Figure 6). At least seven events were detected, which were distributed across the genome (Table 3), which is consistent with some other recent findings from Australia [23,24]. Recently, more and more TuYV (and BrYV) sequences have been analyzed in their complete genome from the Asian region, indicating close relationships between these two virus species [24]. Our result is consistent with these findings, which, in turn, is supported by the fact that the overall p-distance of TuYV and BrYV analyzed here has the same or a lower range (p-distance = 0.092) than the intra-group distance of the sequence groups of BrYV (p-distance = 0.049) or TuYV (p-distance = 0.047) (Supplementary Table S3). Phylogenetic analysis in both Australia and our work showed that all BrYV isolates clade completely within TuYV isolates (Figure 4) [24,32]. Most of the reported TuYV and BrYV isolates are clustered in groups A and B. Group B included TuYV from Europe and Australia, while all BrYV isolates from across Asia in group A had an even higher homology with TuYV from Australia (Figure 4). The latest results therefore suggest that BrYV and TuYV share a common ancestor and may even belong to the same viral species that cannot be separated by a single species. However, further analysis with complex sequences of poleroviruses may provide more clarity on the genetic diversity/similarity within this viral genus.

## 4. Materials and Methods

### 4.1. Plants and Sampling

A mixture of plant tissues from oilseed rape at the 4–6 leaf stage was collected for RNA-seq analysis, including two samples from Yangzhong and three from Nanjing in Jiangsu province. Each sample contained leaves with redness or chlorine symptoms from five individual plants from the respective sampling areas. The samples were then evenly mixed for total RNA isolation and RNA-seq analysis. For the field virus survey, five fields in Yangzhong were randomly selected and labeled A to E. The fields were then mixed equally for total RNA and RNA-seq analysis. For each field, 20–25 samples were collected individually using the five-point method [33]. All plants were randomly sampled whether they had reddened leaves or not, and plants at the edge of the field were avoided.

### 4.2. RNA-Seq and Data Analysis 

RNA extraction and library preparation were performed by Annuoyouda Gene Technology Company (Nanjing, China). Briefly, 3 μg of total RNA was depleted of rRNA using the Ribo-Zero™ Gold Kit. Then, the sequencing library was prepared according to the instructions of the NEB Next Ultra Directional RNA Library Prep Kit for Illumina (NEB, Ispawich, SD, USA). The library was diluted to 1 ng/μL, then insert sizing was performed using Agilent 2100. After quantification analysis using the Bio-RAD KIT iQ SYBR GRN, the HiSeq PE Cluster Kit v4-cBot-HS (Illumia, San Diego, CA, USA) was used to generate clusters on the cBot, and pair-end sequencing was performed on the Hiseq sequencing platform (Illumina, San Diego, CA, USA). A total of 17 G of data was generated with 150 bp paired-end reads. The linker sequence was removed from the raw data using Trimmomatic, and we obtained high-quality, clean reads for subsequent analysis. Next, we downloaded BrYV-AJS (accession number HQ388350), BrYV-BJS (accession number HQ388351) and BrYV-CBJ (accession number KF015269) from NCBI as BrYV references. Hisat2 was used to match the clean reads with the references and Picard to remove sequence repeats and sort bam files [34]. The bedtools bamtofastq command was used to extract the reads from the bam file, and spades was used to assemble the framework.

### 4.3. Phylogenetic Tree Construction, Recombinant Analysis

BrYV-lnc, BrYV-814NJLH and BrYV-NJ13 were used for phylogenomic analysis of the complete genome sequence. Meanwhile, 77 complete virus genome sequences were downloaded from the NCBI database for further analysis (Supplementary Table S2). They mainly contained 18 BrYV, 55 TuYV and four other sequences used as outgroup, including ToYV, BWYV, PLRV and BMYV. We then used MEGA X [35] to build a phylogenetic tree based on the maximum likelihood method. The General Time Reversible model with 1000 bootstrap repeats was used to calculate the result. The amino acid sequences of 24 viruses in each group were aligned using MEGA X, and the pairwise identity was represented as a matrix using TBtools [35,36]. RDP4 was used for recombination analysis [25]. The pairwise distances between 80 BrYV and TuYV sequences were calculated and used to construct a phylogenetic tree using MEGA X. Then, the sequences whose nucleotide sequence identity was less than 70% were discarded, and an alignment was created with the remaining sequences. The preliminary alignment was refined by deleting or moving sequences that could not be aligned and realigning them with the subsections. Finally, the alignment file was opened in RDP4, and the RDP, GENECONV, BootScan, MaxChi, Chimaera, SiScan, and 3Seq methods were used to scan the corresponding alignment according to the RDP user manual [25].

### 4.4. Virus Detection and Cloning

Reverse transcription polymerase chain reaction (RT-PCR) was used for virus detection. All primers used in this study are listed in Supplementary Table S4. Total RNA was isolated using RNA-easyTM Isolation Reagent (Vazyme, R701, Nanjing, China) according to the instructions. Three micrograms of total RNA was used with the HiScript 1st Strand cDNA Synthesis Kit (Vazyme, R111, Nanjing, China). The primer design and amplification conditions of BrYV strains, TuYV, CMV, CABYV and TuMV were adopted according to previous reports [5,37,38,39]. The plasmids containing PCR fragments of isolate BrYV-A (accession number OP779860) and BrYV-B (accession number OP779861) were used as positive controls for the detection of BrYV in this study. For the detection of BWYV-ST9, GMMV and CABYV-AR, new primers were designed according to the assembled fragments (Supplementary Table S1). A total reaction mixture of 25 μL contained 3 μg RNA, 1× PCR buffer minus Mg^2+^, dNTP mixture 0.2 mM, MgCl_2_ 1.5 mM, 2 μL cDNA, 1 unit Taq DNA polymerase (Invitrogen, 18038) and 0.5 μM forward and reverse primers. The reaction mixture was first incubated at 94 °C for 5 min, followed by 32 cycles of denaturation at 94 °C for 45 s, annealing at 57 °C for 30 s and extension at 72 °C for 45 s. The final extension at 72 °C for 10 min was performed. The PCR product was tested on a 1.2% agar gel.

To construct the full-length BrYV clone, three pairs of specific primers were designed to match the assembled BrYV-lnc. Specifically, the first 2.6 kb fragment at the 5′ end of the genome was amplified using primers BrYV-1F and 1R. The middle 2 kb fragment was amplified using primers BrYV-2F and 2R, and the remaining 3′-terminal 1 kb fragment was cloned using primer pairs BrYV-3F and 3R. After purification by gel recovery (Qiagen, 28704, Hilden, Germany), the PCR products were inserted into pEASY-T plasmind and transformed into Trans1-T1 competent cells (Transgene, CT111, Beijing, China). The positive clones were then sequenced.

### 4.5. Leaf Image Capture, Color Quantification and Correlation Analysis

The image of the single leaf was taken with a digital camera. Segmentation of the leaf from the background was then carried out in the following steps. First, the original RGB images were converted to HSV color images and the L-color channel was used to determine a binary threshold for segmenting the leaf. Second, the threshold to distinguish the leaf from the background was determined by visual search and the threshold was used to create the mask. Finally, the mask is used to separate the leaf. Then, the segmented leaf images were used to calculate color indices from the RGB color mode. The indices were calculated as follows:

G_norm = G/(R + G + B)(1)

VI_green = (G − R)/(G + R)(2)

R, G and B are the mean values of the red, green and blue channels in a digital image. G_norm stands for the normalized G values, VI_green is a vegetation index often used in ecological studies. It has been reported that these two color indices are correlated with leaf green [40]. Statistical analysis was performed using the R version 4.1.0 software environment. Pearson correlation analysis was performed with the R package.

## 5. Conclusions

We have shown in this paper that BrYV in Jiangsu, China, causes a disease similar to that caused by TuYV in oilseed rape in Europe or elsewhere in the world. We found that the disease symptoms cause color abnormalities such as red, purple leaves or purple stem initiation on oilseed rape, resulting in severe crop damage in the field. Based on the study of genetic diversity through phylogenetic, recombinant events and pairwise distance analyses, the close relationship between BrYV and TuYV has been demonstrated. The result of our and other studies from Europe and Australia suggest that BrYV could be considered as the Asian variant of the TuYV. As TuYV is the most widespread viral pathogen and severely affects oilseed rape yields in Europe and Australia, the assessment of BrYV infection could predict a similar impact on oilseed rape yields in China. Therefore, in addition to studying virus occurrence and monitoring natural reservoirs, control of the aphid vector in oilseed rape fields is crucial. Nevertheless, resistance breeding in oilseed rape against BrYV should be a prospective and sustainable approach for yield stability of oilseed rape in China.

## Figures and Tables

**Table 1:** The incidence of virus diseases in the field in oilseed rape in Yangzhong.

Fields	Total Samples	BrYV-A	BrYV-B	AB Mix	BrYV	BrYV Incidence (%)	TuYV	TuYV Incidence (%)	TuMV	TuMV Incidence (%)
A	20	3	0	1	4	20.00	0	0.00	11	55.00
B	24	2	1	2	5	20.83	1	4.17	8	33.33
C	23	1	2	3	6	26.09	0	0.00	3	13.04
D	19	0	4	5	9	47.37	0	0.00	3	15.79
E	17	7	1	1	9	52.94	5	29.41	0	0.00
Total	103	13	8	12	33	32.04	6	5.83	25	24.27

**Table 2:** Pairwise amino acid identity of BrYV and TuYV coding protein.

	P0 (%)	P1 (%)	P2 (%)	P3 (%)	P3a (%)	P4 (%)	P5 (%)
BrVY	91.97~100	88.3~100	97.3~100	94.06~100	82.22~100	86.89~100	51.55~100
Br to Tu-FL1	86.75~89.96	89.31~91.6	96.22~97.84	95.05~98.02	86.67~97.78	90.16~96.57	46.84~77.48
814NJLH to Br	94.38~100	89.07~99.67	97.84~100	96.04~100	84.44~100	89.62~100	57.11~100
NJ13 to Br	93.17~100	88.65~99.67	97.84~100	96.04~100	84.44~100	89.62~100	56.91~99.75

**Table 3:** The list of recombinant events identified in this study. Major and minor parent stand for the parent contributing the minor and major part of the sequence, respectively. The recombination site indicates the position of the breakpoint in the corresponding recombinant sequence. “R”, “G”, “B”, “M”, “C”, “S” and “T” are the abbreviations for the detection methods of RDP, GENECONV, BOOTSCAN, MAXCHI, CHIMAERA, SISCAN and 3SEQ according to RDP4 (Recombination Detection Programme version 4) [25].

Events	Recombinant	Major Parent	Minor Parent	Recombination Site		*p*-Values of Different Detection Method						
				Begin	End	R	G	B	M	C	S	T
1	TuYV-C20A	TuYV-Br12	TuYV-MJ11-1	1492	2663	1.99 × 10^−19^	1.70 × 10^−15^	2.00 × 10^−18^	1.15 × 10^−14^	7.92 × 10^−16^	6.92 × 10^−16^	3.89 × 10^−15^
2	TuYV-C2016a	TuYV-YK12-1	TuYV-5509	2668	5654	6.43 × 10^−47^	2.37 × 10^−43^	3.23 × 10^−48^	4.85 × 10^−19^	4.97 × 10^−22^	7.62 × 10^−37^	6.64 × 10^−51^
3	TuYV-MK111	TuYV-MK107	BrYV-NJ13	3368	5508	5.01 × 10^−60^	2.81 × 10^−59^	1.67 × 10^−63^	2.72 × 10^−36^	2.75 × 10^−30^	1.12 × 10^−53^	1.27 × 10^−13^
4	TuYV-MJ11-2	TuYV-MJ11-3	TuYV-JKI	3316	4277	1.08 × 10^−49^	2.58 × 10^−50^	5.99 × 10^−47^	2.74 × 10^−20^	1.83 × 10^−20^	2.60 × 10^−21^	5.11 × 10^−13^
5	TuYV-1740	TuYV-MK107	TuYV-5510	3496	5624	9.20 × 10^−63^	2.12 × 10^−56^	6.52 × 10^−64^	5.45 × 10^−33^	5.02 × 10^−22^	6.61 × 10^−58^	1.27 × 10^−13^
6	TuYV-5514b	TuYV-MK113	BrYV-CR	3842	4872	3.88 × 10^−70^	4.34 × 10^−68^	5.92 × 10^−68^	3.37 × 10^−36^	2.90 × 10^−36^	8.55 × 10^−41^	1.48 × 10^−13^
7	TuYV-P6_2	TuYV-Salzlandkreis-1	BrYV-R3b	3878	5628	1.33 × 10^−101^	6.41 × 10^−76^	3.33 × 10^−94^	9.24 × 10^−44^	2.92 × 10^−13^	4.18 × 10^−69^	9.53 × 10^−56^

## Data Availability

Data are included in the article or Supplementary Materials. The RNA-Seq sequences of the viruses have been deposited in the NCBI database under accession number PRJNA782664. The genome sequences of BrYV-lnc, BrYV-814NJLH and BrYV-NJ13 have been deposited in the NCBI database under accession numbers ON804808, ON804809 and ON804810. The sequence of the fragments of BrYV-A and BrYV-B has been deposited in the NCBI database under accession numbers OP779860 and OP779861.

## Supplementary Materials

The following supporting information can be downloaded at: https://www.mdpi.com/article/10.3390/plants12051008/s1, Table S1. Correlation analysis between disease incidence and leaf color value in five fields; Table S2. Full-length nucleotide sequences of 21 BrYV isolates, 55 TuYV isolates and 4 outgroup species (BWYV, BMYV, ToYV and PLRV) analyzed in this study; Table S3: Genetic distances calculated within groups of BrYV and TuYV isolates; Table S4. The primer sequences used in this study; Figure S1: Three types of plants with different symptoms after BrYV infection in the field on oilseed rape; Figure S2. The distribution of the leaf color value in five fields; Figure S3. Pairwise analysis of BrYV coding proteins; Figure S4. The RDP method plots of 7 recombinants among BrYV and TuYV isolates; Figure S5. The electrophoretogram of RT-PCR analysis against BrYV.

## References

[1] Stevens M., McGrann G.R.D., Clark B. (2008). Turnip yellows virus (syn Beet western yellows virus): An emerging threat to European oilseed rape production?. HGCA Res. Rev..

[2] Jones R.A.C., Coutts B.A., Hawkes J. (2007). Yield-limiting potential of Beet western yellows virus in *Brassica napus*. Aust. J. Agric. Res..

[3] Sõmera M., Fargette D., Hébrard E., Sarmiento C. (2021). ICTV Report Consortium. ICTV Virus Taxonomy Profile: *Solemoviridae* 2021. J. Gen. Virol..

[4] Hackenberg D., Asare-Bediako E., Baker A., Walley P., Jenner C., Greer S., Bramham L., Batley J., Edwards D., Delourme R. (2020). Identification and QTL mapping of resistance to Turnip yellows virus (TuYV) in oilseed rape, *Brassica napus*. Theor. Appl. Genet..

[5] Zhang X.Y., Peng Y.M., Xiang H.Y., Wang Y., Li D.W., Yu J.L., Han C.G. (2022). Incidence and prevalence levels of three aphid-transmitted viruses in crucifer crops in China. J. Integr. Agric..

[6] Kamitani M., Nagano A.J., Honjo M.N., Kudoh H. (2016). RNA-Seq reveals virus-virus and virus-plant interactions in nature. FEMS Microbiol. Ecol..

[7] Lim S., Yoo R.H., Igori D., Zhao F., Kim K.H., Moon J.S. (2015). Genome sequence of a recombinant brassica yellows virus infecting Chinese cabbage. Arch. Virol..

[8] Xiang H.Y., Dong S.W., Shang Q.X., Zhou C.J., Li D.W., Yu J.L., Han C.G. (2011). Molecular characterization of two genotypes of a new polerovirus infecting brassicas in China. Arch. Virol..

[9] Gray S., Cilia M., Ghanim M. (2014). Circulative, “nonpropagative” virus transmission: An orchestra of virus-, insect-, and plant-derived instruments. Adv. Virus Res..

[10] Zhang X.Y., Xiang H.Y., Zhou C.J., Li D.W., Yu J.L., Han C.G. (2014). Complete genome sequence analysis identifies a new genotype of brassica yellows virus that infects cabbage and radish in China. Arch. Virol..

[11] Yoshida N., Tamada T. (2019). Host range and molecular analyses of Beet leaf yellowing virus, Beet western yellows virus-JP and Brassica yellows virus in Japan. Plant Pathol..

[12] Taliansky M., Mayo M.A., Barker H. (2003). Potato leafroll virus: A classic pathogen shows some new tricks. Mol. Plant Pathol..

[13] Derrien B., Baumberger N., Schepetilnikov M., Viotti C., De Cillia J., Ziegler-Graff V., Isono E., Schumacher K., Genschik P. (2012). Degradation of the antiviral component ARGONAUTE1 by the autophagy pathway. Proc. Natl. Acad. Sci. USA.

[14] Li Y.Y., Sun Q., Zhao T.Y., Xiang H.Y., Zhang X.Y., Wu Z.Y., Zhou C.J., Zhang X., Wang Y., Zhang Y.L. (2019). Interaction between brassica yellows virus silencing suppressor P0 and plant SKP1 facilitates stability of P0 in vivo against degradation by proteasome and autophagy pathways. New Phytol..

[15] Prüfer D., Kawchuk L., Monecke M., Nowok S., Fischer R., Rohde W. (1999). Immunological analysis of potato leafroll luteovirus (PLRV) P1 expression identifies a 25kDa RNA-binding protein derived via P1 processing. Nucleic Acids Res..

[16] van der Wilk F., Verbeek M., Dullemans A.M., van der Heuvel J.F.J.M. (1997). The genome-linked protein of Potato leafroll virus is located downstream of the putative protease domain of the ORF1 product. Virology.

[17] Smirnova E., Firth A.E., Allen Miller W., Scheidecker D., Brault V., Reinbold C., Rakotondrafara A.M., Chung B.Y.W., Ziegler-Graff V. (2015). Discovery of a small non-AUG-initiated ORF in poleroviruses and luteoviruses that is required for long-distance movement. PLoS Pathog..

[18] Schmitz J., Stussi-Garaud C., Tacke E., Prüfer D., Rohde W., Rohfritsch O. (1997). In situ localization of the putative movement protein (pr17) from potato leafroll lutevirus (PLRV) in infected and transgenic potato plants. Virology.

[19] Zhang X.Y., Zhao T.Y., Li Y.Y., Xiang H.Y., Dong S.W., Zhang Z.Y., Wang Y., Li D.W., Yu J.L., Han C.G. (2018). The conserved proline18 in the Polerovirus P3a is important for brassica yellows virus systemic infection. Front. Microbiol..

[20] Xu Y., Ju H.J., Deblasio S., Carino E.J., Johnson R., MacCoss M.J., Heck M., Allen Miller W., Gray S.M. (2018). A stem-loop structure in Potato leafroll virus open reading frame 5 (ORF5) is essential for readthrough translation of the coat protein ORF stop codon 700 bases upstream. J. Virol..

[21] Byrne M.J., Steele J.F.C., Hesketh E.L., Walden M., Thompson R.F., Lomonossoff G.P., Ranson N.A. (2019). Combining transient expression and Cryo-EM to obtain high-resolution structures of Luteovirid particles. Structure.

[22] Chen X.R., Wang Y., Zhao H.H., Zhang X.Y., Wang X.B., Li D.W., Yu J.L., Han C.G. (2018). Brassica yellows virus’ movement protein upregulates anthocyanin accumulation, leading to the development of purple leaf symptoms on *Arabidopsis thaliana*. Sci. Rep..

[23] Umar M., Farooq T., Tegg R.S., Thangavel T., Wilson C.R. (2022). Genomic characterisation of an isolate of Brassica yellows virus associated with brassica weed in Tasmania. Plants.

[24] Filardo F., Nancarrow N., Kehoe M., McTaggart A.R., Congdon B., Kumari S., Aftab M., Trębicki P., Rodoni B., Thomas J. (2021). Genetic diversity and recombination between turnip yellows virus strains in Australia. Arch. Virol..

[25] Martin D.P., Murrell B., Golden M., Khoosal A., Muhire B. (2015). RDP4: Detection and analysis of recombination patterns in virus genomes. Virus Evol..

[26] Abraham A.D., Menzel W., Lesemann D.E., Varrelmann M., Vetten H.J. (2006). Chickpea chlorotic stunt virus: A new polerovirus infecting cool-season food legumes in Ethiopia. Virology.

[27] Buxton-Kirk A., Adams I., Frew L., Ward R., Kelly M., Forde S., Skelton A., Harju V., Baucas N.S., Bas-Ilan M.A.G. (2021). First report of Turnip yellows virus in cabbage in the Philippines. New Dis. Rep..

[28] Gaafar Y.Z.A., Ziebell H. (2019). Two divergent isolates of turnip yellows virus from pea and rapeseed and first report of turnip yellows virus-associated RNA in Germany. Microbiol. Resour. Announc..

[29] Milošević D., Ignjatov M., Nikolić Z., Stanković I., Bulajić A., Marjanović-Jeromela A., Krstić B. (2016). The presence of Turnip yellows virus in oilseed rape (*Brassica napus* L.) in Serbia. Pestic. Phytomed..

[30] Slavíková L., Ibrahim E., Alquicer G., Tomašechová J., Šoltys K., Glasa M., Kundu J.K. (2022). Weed hosts represent an important reservoir of turnip yellows virus and a possible source of virus introduction into oilseed rape crop. Viruses.

[31] Nancarrow N., Aftab M., Hollaway G., Rodoni B., Trębicki P. (2022). Symptomless turnip yellows virus infection causes grain yield loss in lentil and field pea: A three-year field study in south-eastern Australia. Front. Plant Sci..

[32] Umar M., Tegg R.S., Farooq T., Thangavel T., Wilson C.R. (2022). Abundance of Poleroviruses within Tasmanian pea cops and surrounding weeds, and the genetic diversity of TuYV isolates found. Viruses.

[33] Fang Z.D., Zhang H.G. (1998). The investigation of plant diseases. Research Methods of Plant Pathology.

[34] Kim D., Paggi J.M., Park C., Bennett C., Salzberg S.L. (2019). Graph-based genome alignment and genotyping with HISAT2 and HISAT-genotype. Nat. Biotechnol..

[35] Kumar S., Stecher G., Li M., Knyaz C., Tamura K. (2018). MEGA X: Molecular evolutionary genetics analysis across computing platforms. Mol. Biol. Evol..

[36] Chen C., Chen H., Zhang Y., Thomas H.R., Xia R. (2020). Tbtools: An integrative toolkit developed for interactive analyses of big biological data. Mol. Plant.

[37] Zhang X.Y., Peng Y.M., Wang Y., Zhang Z.Y., Li D.W., Yu J.L., Han C.G. (2016). Simultaneous detection and differentiation of three genotypes of Brassica yellows virus by multiplex reverse transcription-polymerase chain reaction. Virol. J..

[38] Wei T.Y., Zhang C.W., Hou X.L., Sanfaçon H., Wang A.M. (2013). The SNARE protein Syp71 is essential for Turnip mosaic virus infection by mediating fusion of virus-induced vesicles with chloroplasts. PLoS Pathog..

[39] Shang Q.X., Xiang H.Y., Han C.G., Li D.W., Yu J.L. (2008). Partial sequence analysis of two isolates of Cucurbit aphid-borne yellows virus from Hubei and Yunnan in China. Acta Phytopathol. Sin..

[40] Wang Y., Wang D.J., Shi P.H., Omasa K. (2014). Estimating rice chlorophyll content and leaf nitrogen concentration with a digital still color camera under natural light. Plant Methods.

