# Supplementary material for: Genetic Diversity Analysis of Brassica Yellows Virus Causing Aberrant Color Symptoms in Oilseed Rape
Source: Plants (Basel). 2023 Feb 23;12(5):1008. doi: 10.3390/plants12051008 (PMC10005696; doi:10.3390/plants12051008)
Supplement: Supplementary file 1 [file plants-12-01008-s001.zip › plants-2211250-supplementary.pdf]

Supplementary materilas

Supplementary Table S1. Correlation analysis between disease incidence and leaf colour value in five fields

Disease Incidence	G norm		VI_green	
	R^2	p-value	R^2	p-value
BrYV%	0.09	0.63	0.10	0.606
TuMV%	0.01	0.882	0.01	0.899
Virus	0.33	0.31	0.49	0.191

Supplementary Table S2. Full-length nucleotide sequences of 21 BrYV isolates, 55 TuYV isolates and 4 outgroup species (BWYV, BMYV, ToYV and PLRV) analyzed in this study.

**Host**	**Location**	**Virus isolate**	**Accession number**
Cicer arietinum	Australia	TuYV-MK111	MT586573
Cicer arietinum	Australia	TuYV-5510	MT586572
Brassica napus	Australia	TuYV-5514b	MT586576
Lens culinaris	Australia	TuYV-L31_4	MT586574
Pisum sativum	Australia	TuYV-P6_2	MT586575
Cicer arietinum	Australia	TuYV-1740	MT586571
Brassica oleracea	Australia	TuYV-MJ11-2	LR584025
Cicer arietinum	Australia	TuYV-YK12-1	LR584027
Brassica napus	Australia	TuYV-MK107	MT586590
Brassica napus	Australia	TuYV-C2016a	MT586585
Lens culinaris	Australia	TuYV-MK113	MT586589
Brassica napus	Australia	TuYV-MK102	MT586577
Sinapis arvensis	Australia	TuYV-5248	MT586581
Brassica napus	Australia	TuYV-MK104	MT586578
Pisum sativum	Australia	TuYV-P5_8	MT586586
Cicer arietinum	Australia	TuYV-BL13-1	LR584020
Brassica napus	Australia	TuYV-BG12-1	LR584019
Brassica napus	Australia	TuYV-C21A	MT586582
Pisum sativum	Australia	TuYV-MK109	MT586579
Brassica napus	Australia	TuYV-5512b	MT586580
Brassica napus	Australia	TuYV-WA-1_Canola	LR584021
Brassica napus	Australia	TuYV-MK106	MT586584
Pisum sativum	Australia	TuYV-MK103	MT586583
Brassica napus	Australia	TuYV-5509	MT586587
Brassica napus	Australia	TuYV-C2016b	MT586588
Raphanus raphanistrum	Australia	TuYV-MJ11-3	LR584026
Brassica napus	Australia	TuYV-C20A	MT586597
Brassica oleracea	Australia	TuYV-MJ11-1	LR584024
Beta vulgaris	Australia	TuYV-Br12	MT586598
Brassica napus	Australia	TuYV-5514a	MT586594
Brassica napus	Australia	TuYV-5513	MT586592
Brassica napus	Australia	TuYV-5512a	MT586593
Brassica napus	Australia	TuYV-5414	MT586591
Brassica napus	Australia	TuYV-MK105	MT586595
Diuris penduncculata	Australia	TuYV-WA-1	JQ862472
Cicer arietinum	Australia	TuYV-5511	MT586596
Chinese cabbage	China	BrYV-C-Haidian	KF015269
Raphanus sativus	China	BrYV-CR	JN015068
Brassica napus	China	BrYV-BJS	HQ388351
Brassica napus	China	BrYV-China	KY310572

Brassica napus	China	BrYV-ABJ	NC_016038
Nicotiana tabacum	China	BrYV-Anhui	MF314820
Brassica campestris	China	BrYV-AJS	HQ388350
Nicotiana tabacum	China	BrYV-NtabQJ	MK057527
Nicotiana tabacum	China	TuYV-Anhui	KR706247
Nicotiana tabacum	China	ToYV-Shandong	MK751486
Brassica napus	China	BrYV-lnc	ON804808
Brassica napus	China	BrYV-BBJ	HQ388349
Brassica napus	China	BrYV-814NJLH	ON804809
Brassica napus	China	BrYV-NJ13	ON804810
Arachis pintoi	Colombia	TuYV-Pintoi-VarA	KU726090
Arachis pintoi	Colombia	TuYV-Pintoi-VarB	KU726091
Lactuca sativa	France	TuYV-FL1	NC003743
Pisum sativum	Germany	TuYV-Salzlandkreis-2_16	MN497803
Pisum sativum	Germany	TuYV-Kreis_Stormarn_16	MN497808
Pisum sativum	Germany	TuYV_Landkreis_Meissen_17	MN497810
Pisum sativum	Germany	TuYV-Muenster_17	MN497806
Pisum sativum	Germany	TuYV-Muenster_18	MN497807
Pisum sativum	Germany	TuYV-Salzlandkreis-2_18	MN497804
Pisum sativum	Germany	TuYV-Salzlandkreis-1_18	MN497802
Pisum sativum	Germany	TuYV-JKI_29344	MK450520
Pisum sativum	Germany	TuYV-Landkreis_Meissen_18	MN497811
Pisum sativum	Germany	TuYV-Salzlandkreis-1_17	MN497801
Raphanus raphanistrum	Germany	TuYV-JKI_29345	MK450519
Pisum sativum	Germany	TuYV-Muenster_16	MN497805
Pisum sativum	Germany	TuYV-Salzlandkreis-1_16	MN497800
Pisum sativum	Germany	TuYV-Landkreis_Meissen_16	MN497809
Raphanus sativus	Japan	BrYV-R3b	LC428363
Brassica rapa	Japan	BrYV-CC1	LC428358
Brassica oleracea	Japan	BrYV-CD9	LC428362
Sinapis alba	Japan	BrYV-WN1	LC428359
Brassica rapa	Japan	BrYV-TO3	LC428360
Raphanus sativus	Japan	BrYV-R40	LC428365
Raphanus sativus	Japan	BrYV-RT8	LC428364
Brassica napus	Japan	BrYV-NAP	LC428361
Brassica oleracea	South_Africa	TuYV-Bo10/12	KU198395
Chinese cabbage	South_Korea	BrYV-CS	KF923236
Beta vulgaris	USA	BWYV	NC_004756
Solanum tuberosum	USA	PLRV	NC_001747
Beta vulgaris	USA	BMYV	NC_003491

Supplementary Table S3: Genetic distances calculated within groups of BrYV and TuYV isolates

Method	P-distance
Overall mean distance	0.092
Within groups mean distance: BrYV	0.049
Within groups mean distance: TuYV	0.047
Between groups mean distance: BrYV vs TuYV	0.0031
Net between groups mean distance: BrYV vs TuYV	0.0021

Supplementary Table S4. The primer sequences used in this study

Primer name	Sequence (5' → 3')	Reference
BrYV 1F	AGAAAAGAAACCAGGAGGGAATC	In this study
BrYV 1R	AATGTTCCTCCTCGGGTTGC	In this study
BrYV 2F	CCCCGAAGCAGAACTAAAGA	In this study
BrYV 2R	GGAAATCAAGCCCAACTCCA	In this study
BrYV 3F	AGCGTGGACAGATCGTGATA	In this study
BrYV 3R	GAGTGCCGTAGGGATTTCTC	In this study
CABYV AR 2299	ACTACATGGTCACTAGCCTCCG	In this study
CABYV AR 2911	AACAATCAACCTCGCTCACG	In this study
BWYV ST9 2017	ACTGTCGAGTAAAGCGGCAA	In this study
BWYV ST9 2449	CCGGTGTATACGAGACTGGC	In this study
GMMV-2201	AGTGGGACCATTAGCCTCG	In this study
GMMV-2900	ATATGCCCTGGGATTCGTG	In this study
TuMV-F	TGGCTGATTACGAACTGACG	Wei *et al.*, 2013
TuMV-R	CTGCCTAAATGTGGGTTTGG	Wei *et al.*, 2013
TuYV-F	AAGAGGCTTGCCCCTTCCTG	Zhang *et al.*, 2016
TuYV-R	AACCAAATCCGGTGTTGGAT	Zhang *et al.*, 2016
BrYA484F	TACTTGGACTAGAGATGCTGAAAG	Zhang *et al.*, 2016
BrY761R	AGACCGAAGAGCTGAAAAGG	Zhang *et al.*, 2016
BrYB88F	CCTCCACCCAAAACAAGTAT	Zhang *et al.*, 2016
BrYC257F	CGAGTTTCCGTACTTGTTG	Zhang *et al.*, 2016
CMV-F	TGTCCRACYGTBAGTAGGGT	Zhang *et al.*, 2021
CMV-R	CGAAGAAACCTAGGAGATGG	Zhang *et al.*, 2021
CABYV-2715	GACTGCTCCGGTTTTGACTGG	Shang *et al.*, 2008
CABYV-4100	CGTCTACCTATTTCGGGT	Shang *et al.*, 2008

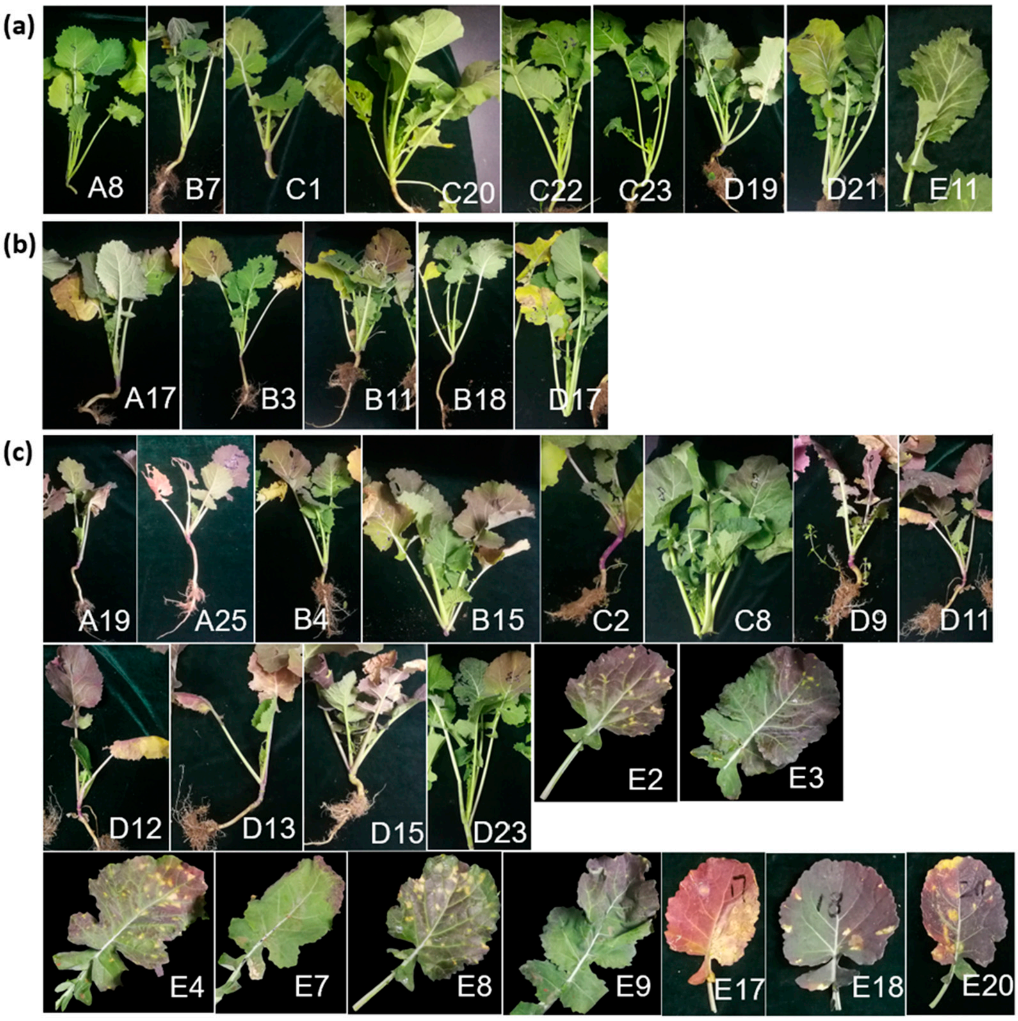

Supplementary Figure S1. Three types of plants with different symptoms after BrYV infecting in field on oilseed rape. (a) displayed the type I plants. These plants exhibited no significant symptoms. (b) was the BrYV infected plants with yellowing leaves in type II. (c) showed the type III BrYV positive plants with remarkable red or purple leaves. The block capitals A, B, C, D and E indicated different samples collected from corresponding fields.

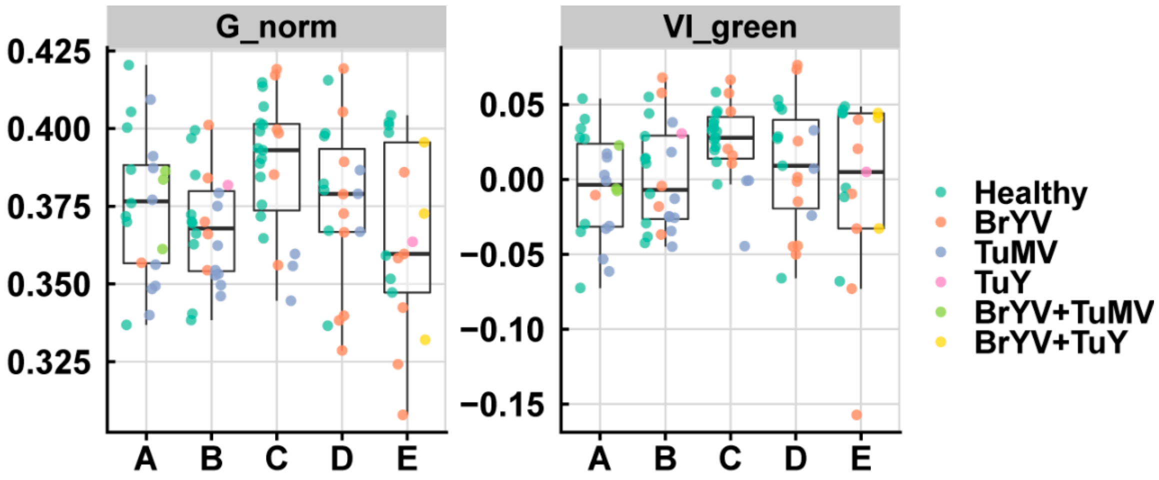

Supplementary Figure S2. The distribution of the leaf color value in five fields. Leaf color values were estimated by G_norm (left) or VI_green (right) parameters. Blue dots indicate healthy plant leaf color values. Orange, cornflowerblue and purple dots suggest the color value of BrYV, TuMV and TuYV infected plant, respectively. Green and yellow dots represent the leaf color values of co-infection of BrYV and TuMV, and BrYV and TuYV, respectively. The block capitals A, B, C, D and E indicated five sampling fields.

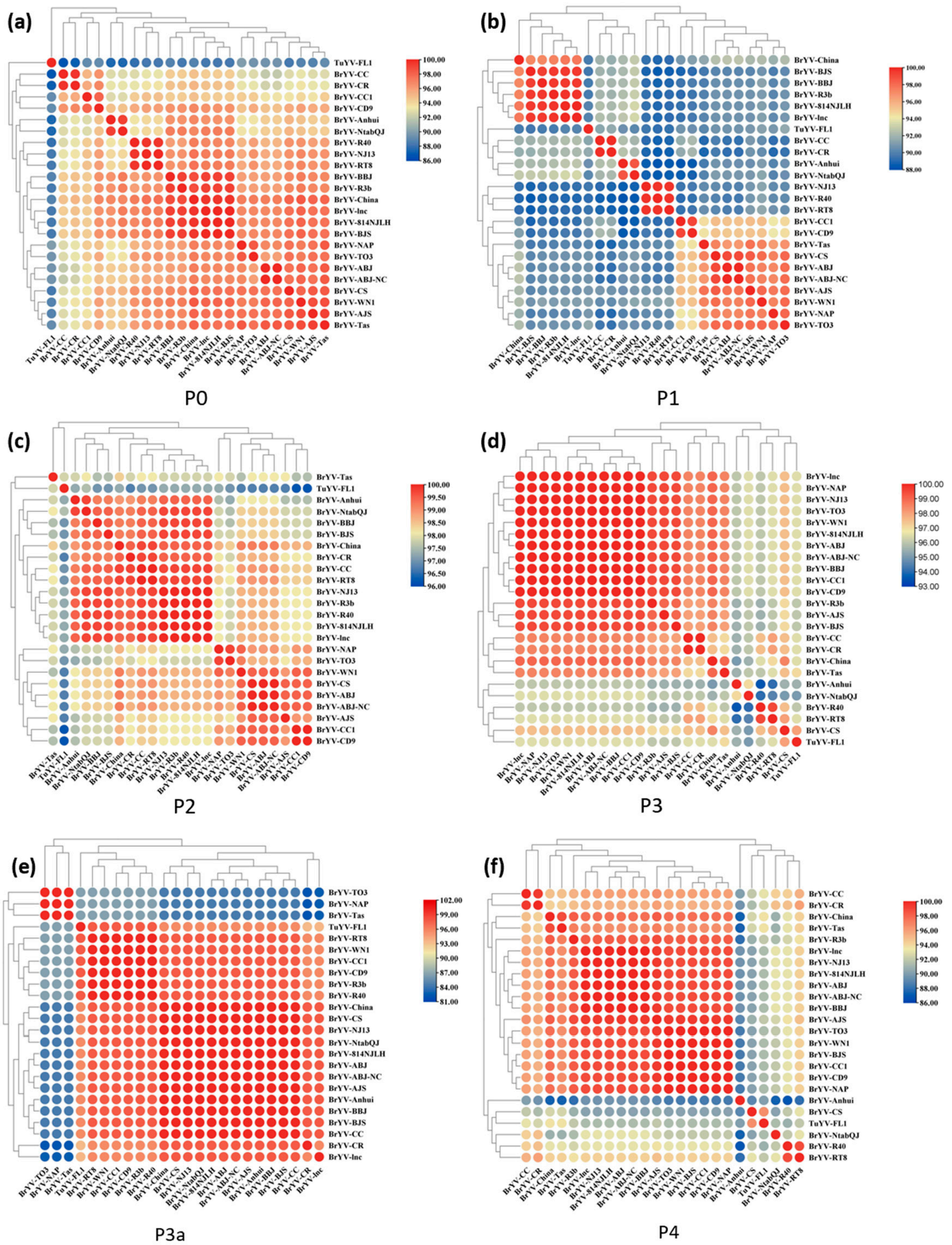

Supplementary Figure S3. Pairwise analysis of BrYV coding proteins. The matrix displayed pairwise amino acid identity among different BrYV and TuYV isolates in (a) P0 protein (RNA silencing suppressor), (b) P1 protein , (c) P2 protein, (d) P3 coat protein, (e) P3a protein and (f) P4 movement protein. All virus isolates information and GenBank accession numbers were depicted in Table S3. The phylogenetic trees based on aligned amino acid sequence of BrYV and TuYV. The identity of each amino acid pairwise was indicated by colored dot.

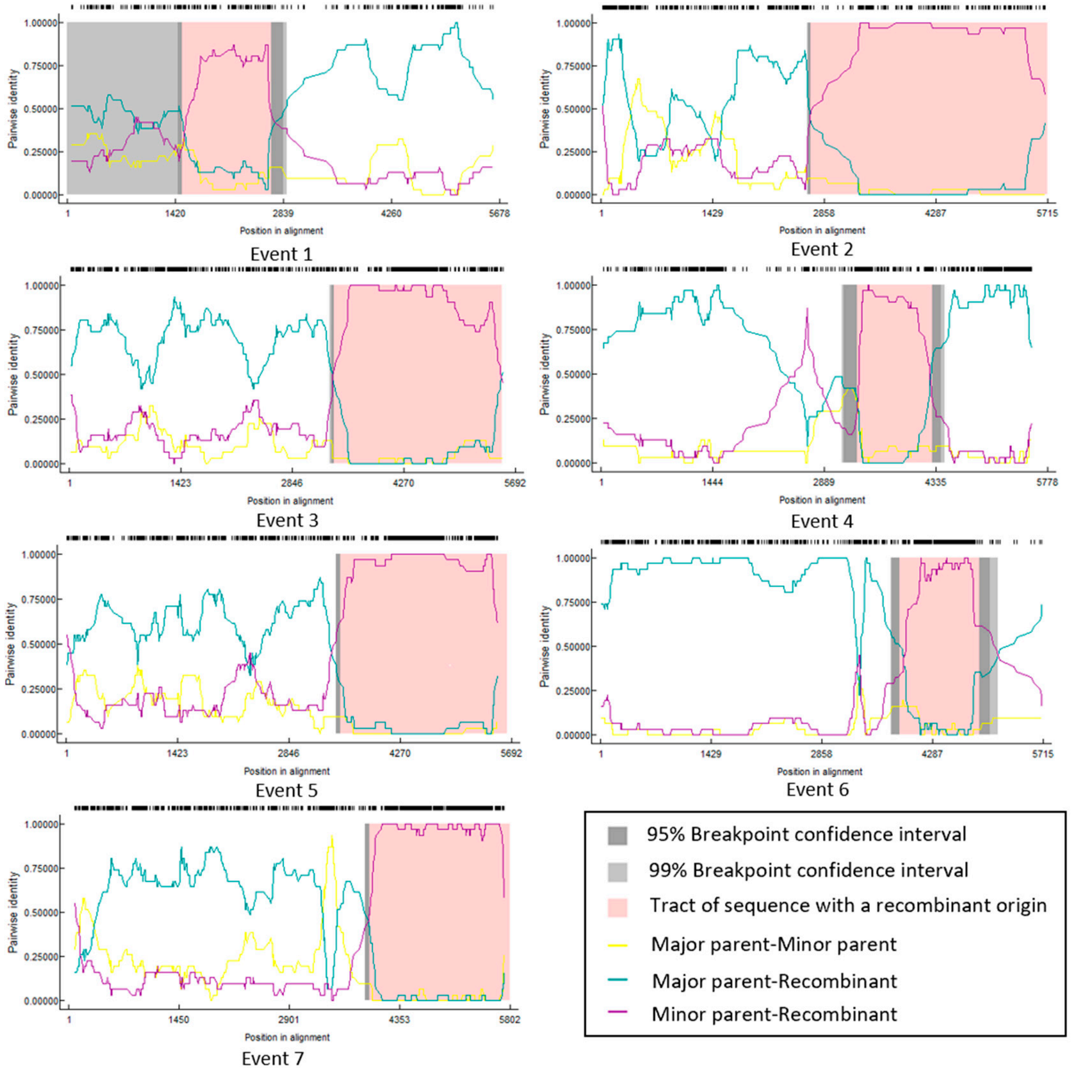

Supplementary Figure S4. The RDP method plots of 7 recombinants among BrYV and TuYV isolates. The detailed information of the seven recombinants was introduced in Table 3. For each recombinant event, the left and right boundaries of the pink area indicated the inferred recombination breakpoint locations. The dark grey and grey areas marked 95% and 99% breakpoint confidence interval, respectively. The x, y axes supplied particular location in the relevant recombinant sequence and the pairwise identity of major parent-minor parent (yellow line), major parent-recombinant (green line) and minor parent-recombinant (purple line). Vertical lines above the plot illustrated positions of the variable nucleotide sites.

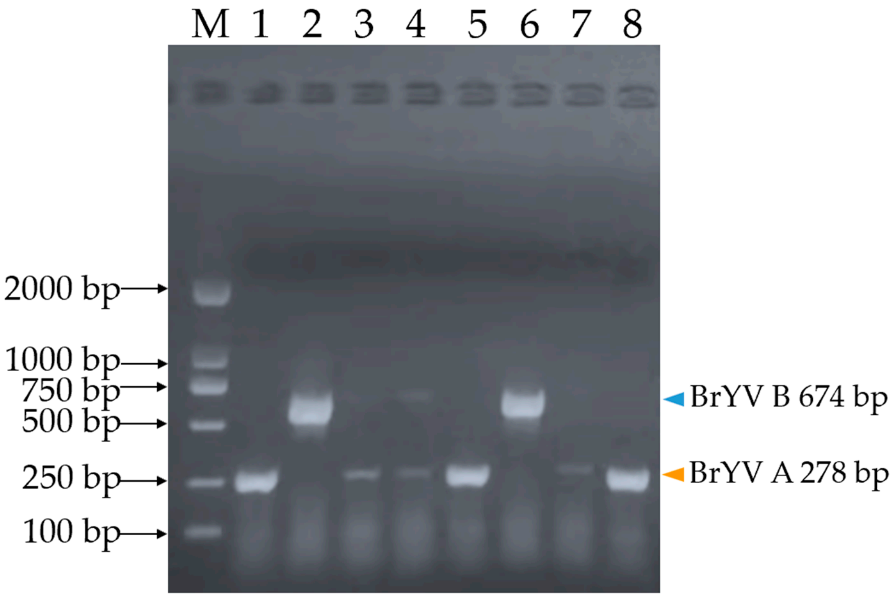

Supplementary Figure S5. The electrophoretogram of RT-PCR analysis against BrYV. Lane M: DNA marker indicated PCR products size ranging from 100 bp to 2000 bp; Lane 1 and 2 were BrYV-A and BrYV-B plasmid control, respectively; Lane 3, 4 and 5 were samples from Chuxiong in Yunnan province; Lane 6, 7 and 8 were samples from Changshun in Guizhou province. The orange triangle with the length of 278 bp suggested BrYV A test band which was amplified by primer pairs of BrYA484F and BrY761R. The blue triangle at around 674 bp indicated BrYV B test band which was detected by BrYB88F and BrY761R.
